# Assessing the applicability of the 2023 international MOGAD panel criteria in real-world clinical settings

**DOI:** 10.1007/s00415-024-12438-6

**Published:** 2024-05-29

**Authors:** Ariel Rechtman, Tal Freidman-Korn, Omri Zveik, Lyne Shweiki, Garrick Hoichman, Adi Vaknin-Dembinsky

**Affiliations:** 1 Department of Neurology and Laboratory of Neuroimmunology and the Agnes-Ginges Center for Neurogenetics, Hadassah-Hebrew University Medical Center, Ein-Kerem, Germany; 2 Faculty of Medicine, Hebrew University of Jerusalem, Jerusalem, Israel; 3 Department of Military Medicine, Faculty of Medicine, Hebrew University of Jerusalem, Jerusalem, Israel; 4 Neurology Department, Multiple Sclerosis and Immunobiology Research, Hadassah Medical Center, Ein-Kerem, POB 12000, 91120 Jerusalem, Israel

**Keywords:** MOGAD, Diagnosis, Volumetry, MOG-IgG

## Abstract

**Introduction:**

Myelin oligodendrocyte glycoprotein antibody-associated disease (MOGAD) is a recently identified demyelinating disorder with a diverse clinical spectrum. Diagnosing MOGAD traditionally relies on clinical judgment, highlighting the necessity for precise diagnostic criteria. Banwell et al. proposed criteria, aiming to refine the diagnostic spectrum. This study evaluates these criteria in a real-life cohort, comparing their performance with clinical judgment and describe the cohort of MOGAD patients.

**Methods:**

This retrospective study, conducted at Hadassah Medical Center, included 88 patients with MOG-IgG antibodies. Patients with a positive or borderline MOG-IgG antibodies by cell-based assay were included. Demographics, clinical and MRI data were recorded. Cases were divided into definite MOGAD and Non-MOGAD groups as determined by the treating physician. We assessed the sensitivity and specificity of the new criteria in comparison to treating physicians’ evaluations. Additionally, we examined clinical differences between the MOGAD and Non-MOGAD groups.

**Results:**

We observed a strong concordance (98%) between the new MOGAD criteria and treating physicians' diagnoses. Clinical disparities between MOGAD and Non-MOGAD groups included lower EDSS scores, normal MRI scans, preserved brain volume, negative OCB results, and distinct relapse patterns. Also, compared to relapsing patients, monophasic MOGAD patients have greater brain volume and a lower age at onset.

**Conclusion:**

The study demonstrates robust accuracy of new MOGAD criteria, emphasizing their potential to enhance diagnostic precision. Treatment response integration into the MOGAD diagnosis is crucial, as it could aid in distinguishing MOGAD from other demyelinating disorders. Distinct clinical profiles highlight the importance of informed decisions in managing MOGAD and similar disorders.

## Introduction

Myelin oligodendrocyte glycoprotein antibody-associated disease (MOGAD) is a recently defined demyelinating disorder affecting the central nervous system, manifesting across all age groups with a broad clinical spectrum encompassing monophasic and relapsing presentations [1, 2]. The protein myelin oligodendrocyte glycoprotein (MOG) is situated on the surface of myelin-forming oligodendrocytes [3]. In MOGAD, the IgG antibody targets the MOG protein, leading to demyelination and neurological symptoms [4, 5]. Recent advancements in the detection of MOG antibodies have contributed significantly to characterizing the MOGAD spectrum [6, 7]. However, the widespread use of MOG antibody testing raises the risk of false positives, emphasizing the importance of cautious interpretation [8].

MOGAD is primarily linked to acute disseminated encephalomyelitis (ADEM) in young children, while adults often present with optic neuritis (ON) and myelitis, with lower prevalence in cases of encephalitis and seizures [9, 10]. In comparison to multiple sclerosis (MS) and neuromyelitis optica spectrum disorders (NMOSD), MOGAD is characterized by a younger age at onset, equal frequency in males and females, and a preference for optic nerve involvement [11, 12].

Approximately 33–45% of adults with MOGAD exhibit brain lesions, with one-third of these lesions located in the infratentorial region, particularly in the brainstem [13–15]. In children, bilateral and sizable brainstem lesions, along with lesions in the deep gray nuclei, are more frequently observed [16, 17]. MOGAD orbital MRI typically shows an edematous, enlarged nerve that can have a short or long segment of T2 hyperintensity [18]. This finding predominantly involves the anterior segments of the optic nerve [18]. Bilateral involvement of the optic nerves on orbital MRI is common [13].

Diagnosing MOGAD has traditionally relied on clinical judgment, supported by serology testing for MOG antibodies. Given its nature as a demyelinating disorder, there is a significant overlap with NMOSD and MS in both clinical presentation and neuroradiology findings [19, 20]. This underscores the need for precise diagnostic criteria for MOGAD to distinguish it from similar conditions.

Banwell et al. recently published criteria for MOGAD, aiming to refine the diagnostic spectrum and improve accuracy [21]. These criteria encompass three main components: (1) a core clinical demyelinating event; (2) a positive IgG MOG antibody test; (3) exclusion of a better diagnosis. This study aims to evaluate these criteria in a real-life cohort of MOGAD patients and compare their performance with the clinical judgment of treating physicians.

## Methods

### Patients

We conducted a single tertiary center retrospective study. Records were extracted from existing medical files of patients who were admitted to the Neurology department at Hadassah Medical Center and/or received follow-up care in neurology outpatient clinics from 2017 to 2023.We identified 88 patients (adults and pediatrics) with MOG IgG ab positive or borderline as described in the recently published criteria for MOGAD, using cell-based indirect immunofluorescence assays [21]. Most samples were taken before the initiation of therapy.

Clinical data, encompassing gender, age, core clinical attack, disease course, magnetic resonance imaging (MRI) features (including lesion distribution and volumetric parameters), oligoclonal bands (OCB) status, and expanded disability status scale (EDSS) at presentation, were systematically recorded and subjected to thorough analysis. Patients were categorized as monophasic if they experienced only one clinical attack and underwent a follow-up exceeding 3 years. Those with a single clinical attack but who had not completed the 3-year follow-up were placed in the "unknown" group.

Cases were divided to MOGAD and Non-MOGAD patients according to treating neuroimmunologist prior to applying the new MOGAD criteria. The new proposed criteria were then applied to the patient cohort by an independent investigator (T.F.K) not involved in the clinical care of the patients or in the clinician determined disease categorization.

### Ethical

The study was approved by Hadassah Medical Organization's Ethics Committee (reference no. HMO-20-0644). Given the study design, the Hadassah Medical Organization's Ethics Committee determined that written consent was not required. We confirm that the data collection was performed in accordance with relevant guidelines and regulations.

### Brain MRI

Brain MRI scans were acquired using the demyelination protocol [22]. T1-weighted images were acquired using MRI scanners at Hadassah Ein Kerem medical center as described previously [23]. Volumetric data were extracted using the MDbrain software, an artificial intelligence-based software tool for volumetric brain analysis, which quantifies volumetric values and codes deviations based on findings from a normal database [23]. Brain volume was extracted only for adult patients.

### Statistical analyses

The evaluation of diagnostic agreement between the treating neuroimmunologist and new MOGAD criteria, quantified through percentage agreement and Cohen's Kappa coefficient. Sensitivity and specificity were calculated to assess the diagnostic accuracy of the new criteria. Additionally, receiver operating characteristic (ROC) analysis was conducted, with the area under the curve (AUC) being determined to evaluate overall diagnostic performance.

Comparative analysis of clinical features between MOGAD and non-MOGAD groups was performed using independent sample t tests for continuous variables and chi-square tests for categorical variables. This approach facilitated the assessment of differences in clinical characteristics such as age at onset, number of relapses, gender, type of relapses, and disease phenotype. The correlation between normalized brain volume and various clinical parameters was analyzed using the Spearman rank correlation test.

All analyses were performed using R statistical software, with a p-value threshold of less than 0.05 set for statistical significance.

## Results

We assembled a cohort comprising 88 patients exhibiting either a positive or borderline MOG IgG antibody result. Among these individuals, 46 were females (52%), and 42 were males (48%). The mean age at onset was 26.44 ± 14.23 years, while the mean EDSS at onset was 1.7 ± 1.73. In terms of disease course, 31 patients experienced a monophasic course (25%), 37 exhibited a relapsing course (42%), and 20 patients had not completed the 3-year surveillance necessary for course classification. Regarding brain MRI results, 47 patients (53%) demonstrated normal findings. OCB status was known for 75 patients, with 59 (79%) testing negative. Among the OCB-positive patients, 10 (63%) had a positive MOG result, and 6 (37%) had a borderline result.

Out of the cohort, the first clinical presentation was ON in 49 patients (56%), with 9 experiencing bilateral ON (18%). Myelitis was observed in 22 patients (25%), while 3 patients presented with simultaneous occurrences of both ON and myelitis (3%). Additionally, eight patients presented with ADEM (9%), two patients with encephalitis (2%), and four were categorized under "other" (5%).

**Fig. 1:**
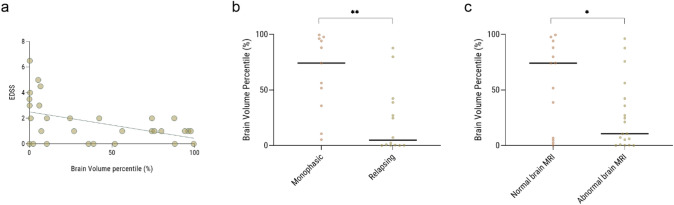
Correlations between brain volume and clinical data of anti-MOG positive patients. **a** There is a significant correlation between normalized brain volume and EDSS (r = -0.46, p = 0.01). **b** Normalized brain volume was significantly higher in patients with a monophasic course compared to those with a relapsing course (64.48 ± 35.10 vs 22.24 ± 30.18, p = 0.004). **c** Normalized brain volume was significantly higher in patients with normal brain MRI scans versus those with abnormal scans (54.74 ± 39.50 vs 26.73 ± 31.14, p = 0.03).

Volumetric data were collected using the MDbrain software from 33 patients with accessible high-quality MRI scans. Among them, 26 (79%) displayed a positive MOG-result, while 7 (21%) showed borderline results. A significant negative correlation was observed between normalized brain volume and EDSS (r = − 0.46, p = 0.01). Additionally, normalized brain volume was significantly higher in patients with a monophasic course compared to those with a relapsing course (64.48 ± 35.10 vs 22.24 ± 30.18, p = 0.004), and in patients with normal brain MRI scans versus those with abnormal scans (54.74 ± 39.50 vs 26.73 ± 31.14, p = 0.03). (Fig. 1a–c) However, no significant correlation was identified between brain volume and age at disease onset, gender, type of relapse, and OCB status.

### Strong concordance observed between the new criteria and physician diagnoses.

**Fig. 2:**
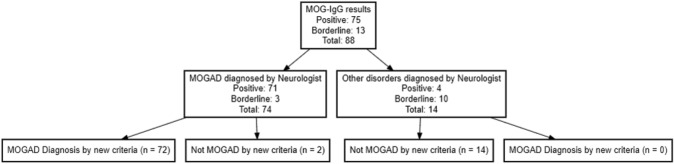
Flowchart comparison of physician diagnoses and new criteria for MOGAD determination

In the comparison of the newly established criteria with the initial diagnoses made by treating physicians, it was found that 86 out of 88 patients maintained consistent diagnoses (Fig. 2). Only two patients initially diagnosed with MOGAD by their physicians did not receive the same diagnosis according to the new criteria.

In the first case, the patient experienced four sequential episodes of optic neuritis. Brain MRI revealed lesions indicative of MS and tested positive for OCBs. Notably, three of these episodes occurred within 1 year following the initiation of interferon beta-1a therapy. The third episode coincided with a tapering down of steroid dosage. During the interferon beta-1a treatment, the patient had clinical exacerbations with the appearance of new lesions on subsequent MRIs. During this period, the patient also tested positive for MOG antibodies at a dilution of 1:100. The concurrent presence of MOG antibodies and the clinical and radiological decline under MS-targeted therapy prompted the consideration of MOGAD by the treating physician. However, the presence of typical MS lesions and positive OCBs, in accordance with updated diagnostic criteria, ultimately supported a diagnosis of MS rather than MOGAD.

In the second case, the patient experienced episodes of diplopia and myelitis. The brain MRI showed multiple lesions and the OCB test was positive. A positive MOG-IgG test (dilution of 1:100) and a favorable response to intravenous immunoglobulins (IVIG) guided her physicians toward the diagnosis of MOGAD. However, the patient demonstrated symptoms and MRI radiological features more in line with MS resulting in a diagnosis of non-MOGAD disease based on the new criteria.

The results demonstrate accuracy rate of 98% for the new MOGAD diagnostic criteria. The overall agreement between the treating neuroimmunologists and the novel MOGAD criteria was exceptional, as evidenced by a Cohen’s kappa value of 0.92.

**Table 1:** Differences in demographics, clinical presentations, radiological findings, and laboratory results between the MOGAD and Non-MOGAD groups

Variable	MOGAD (n = 72)	Other diagnosis (n = 16)	p value
Age at onset (year)	25.575 ± 14.926	30.338 ± 10.045	0.130
EDSS at diagnosis	1.429 ± 1.574	2.846 ± 1.951	0.027
Relapse number	1.639 ± 1.282	2.188 ± 1.377	0.159
Gender	F: 38, M: 34	F: 8, M: 8	1.000
Relapse type	ADEM: 8, both: 7, encephalitis: 2, myelitis: 15, ON: 38, other: 2	Both: 3, myelitis: 3, ON: 6, other: 4	0.021
Disease course	Monophasic: 29, relapsing: 27	Monophasic: 2, relapsing: 10	0.058
MRI brain	Abnormal: 26, normal: 43	Abnormal: 12, normal: 4	0.015
OCB	Negative: 54, positive: 5	Negative: 5, positive: 11	0.000
Brain volume (cm^3^)	46.196 ± 37.271	9.214 ± 15.375	0.001

Compared to clinician assessment, applying the 2023 MOGAD criteria to our institutional cohort yielded a sensitivity of 0.97 and a specificity of 1, leading to a ROC AUC of 0.9865.

According to the new diagnostic criteria, 72 patients were diagnosed with MOGAD, while 16 received a classification labeled as "Other". By considering only items A and B of the criteria among these 16 patients, 8 would have been diagnosed with MOGAD. However, when incorporating the third criterion, which involves the exclusion of alternative diagnoses, these patients did not meet the criteria for MOGAD. A diagnosis of MS was deemed more appropriate for six patients, while NMOSD was considered a more fitting diagnosis for the remaining two.

### Differences between MOGAD and non-MOGAD patients

Upon comparing the two groups, distinct disparities in clinical data surfaced. Individuals diagnosed with MOGAD exhibited significantly lower EDSS scores (1.429 ± 1.574 vs 2.846 ± 1.951, p = 0.027), normal MRI scans at the time of disease onset (64% vs 25%, p = 0.02), preserved brain volume (46.196 ± 37.271 vs 9.214 ± 15.375, p = 0.001), a negative result for OCB (92% vs 31%, p < 0.0001), and a tendency toward a more monophasic disease course (52% vs 17%, p = 0.06). Furthermore, these patients encountered specific types of relapses that differed from those observed in the Non-MOGAD group, with occurrences of ADEM and encephalitis exclusively noted in the MOGAD cohort. (Table 1).

In a focused analysis of the MOGAD group, we noted that there is a trend toward earlier age of onset in monophasic patients (20.88 ± 15.50 vs 28.00 ± 13.64, p = 0.07), with no significant differences observed in terms of EDSS (1.32 ± 1.89 vs 1.23 ± 1.15, p = 0.84), percentage of female patients (48% vs 56%, p = 0.78), MRI brain abnormalities at onset (31% vs 41%, p = 0.63), or OCB positivity (9% vs 9%, p = 1.00) (Table 2). Although the results are not statistically significant when comparing relapse types, monophasic patients tended to experience more myelitis relapses (8/29 28% vs 3/27 11%), while relapsing patients more frequently had relapses involving both myelitis and ON (1/29 3% vs 6/27 22%).

**Table 2:** Differences in demographics, clinical presentations, radiological findings, and laboratory results between the monophasic and relapsing MOGAD patients

Variable	Monophasic (n = 29)	Relapsing (n = 27)	p value
Age at onset (year)	20.88 ± 15.498	28.004 ± 13.644	0.073
EDSS at diagnosis	1.320 ± 1.887	1.227 ± 1.152	0.838
Gender	F: 14, M: 15	F: 15, M: 12	0.782
Relapse type	ADEM: 4, both: 1, myelitis: 8, ON: 15, other: 1	ADEM: 2, both: 6, myelitis: 3, ON: 15, other: 1	0.182
MRI brain	Abnormal: 9, normal: 20	Abnormal: 11, normal: 16	0.632
OCB	Negative: 21, positive: 2	Negative: 20, positive: 2	1.000
Brain volume (cm^3^)	69.870 ± 31.846	29.767 ± 33.537	0.017

## Discussion

The comparative analysis between the newly established diagnostic criteria and the prior diagnoses rendered by treating physicians yielded significant findings, highlighting the effectiveness and reliability of the novel criteria. Demonstrating a high concordance rate of 98%, with 86 out of 88 patients receiving identical diagnoses under both systems, the new criteria showcase robust accuracy. This is further emphasized by the test's impressive sensitivity of 0.96 and perfect specificity of 1, resulting in a ROC area under the curve of 0.9797. The percentage of agreement is further illustrated by Cohen's kappa score, yielding a result of 0.92. These outcomes not only validate the new criteria but also accentuate their potential to enhance diagnostic precision in clinical practice.

The cases where the diagnosis diverged highlight the potential importance of integrating treatment response as a criterion in diagnostic frameworks. Out of the patients initially diagnosed with MOGAD by their clinicians, only two were not diagnosed as such according to the new criteria. Both patients exhibited MRI findings resembling those of MS. In the first case, the observed deterioration following MS treatments and a clinical attack occurring during the tapering down of steroids, and in the second case, the positive response to IVIG, were crucial factors guiding physicians toward a MOGAD diagnosis not supported by the new criteria. Both patients had positive MOGAD antibodies also in the 1:100 dilution. In light of these instances, there is a compelling argument for considering the inclusion of treatment response in diagnostic criteria. Doing so could enhance accuracy and aid in distinguishing MOGAD from other demyelinating disorders.

The reclassification of diagnoses under the new criteria resulted in the identification of 72 patients with MOGAD and 16 with "Other" diagnoses, emphasizing the nuanced nature of these conditions. The subset of patients who might have been classified as MOGAD in the absence of item C in the criteria, involving the exclusion of alternative diagnoses, highlights the critical necessity for precise and tailored diagnostic approaches.

The two patient groups displayed distinctive clinical profiles. The Non-MOGAD group exhibited significantly higher EDSS scores, a more relapsing than monophasic disease course, abnormal MRI scans, reduced brain volume, and positive results for OCB, resembling a clinical course more aligned with MS. In contrast, ADEM and encephalitis were present only in the MOGAD group. This divergence in clinical characteristics holds paramount importance for clinicians, providing vital insights to make informed decisions regarding diagnosis and treatment strategies.

Focusing on MOGAD patients, our findings indicate that monophasic patients tend to have an earlier age of onset compared to those with relapsing disease. Clinical parameters such as EDSS, female:male ration, MRI brain abnormalities at onset, and OCB positivity showed no significant differences between the two groups. Myelitis attacks were more common in monophasic patients, while relapsing patients were more likely to experience both myelitis and ON. Although this trend wasn't statistically significant, it suggests the presence of distinct pathophysiological mechanisms between monophasic and relapsing courses. Further research with larger cohorts and longer follow-up is crucial to validate these findings and develop more effective management strategies for MOGAD.

In accordance with our previous publication, there was a significant correlation between EDSS scores, and normalized brain volume was observed in anti-MOG positive patients, indicating that higher disability scores are associated with greater brain atrophy supporting previous findings that brain atrophy could be a marker of disease progression in demyelinating diseases [23]. Furthermore, relapsing anti-MOG positive patients had significantly lower normalized brain volumes compared to monophasic patients, suggesting that the relapsing nature of the disease might be associated with more extensive brain damage. Similarly, patients with normal initial MRI scans showed higher brain volumes than those with abnormal scans, suggesting that early MRI findings might predict long-term brain health and disease impact.

In conclusion, the findings from this study not only validate the new diagnostic criteria but also illuminate the diverse clinical spectrums within MOGAD and similar neurological disorders. It is important to incorporate treatment response into the diagnostic criteria, as it has the potential to enhance accuracy and assist in differentiating MOGAD from other demyelinating disorders. This enhanced understanding can significantly influence treatment strategies, prognosis, and overall patient care. The study's results encourage ongoing research and refinement of diagnostic tools, ensuring they remain sensitive and specific to the evolving landscape of neurological disorders.
