# Correction to: Assessing the applicability of the 2023 international MOGAD panel criteria in real-world clinical settings

**DOI:** 10.1007/s00415-024-12550-7

**Published:** 2024-09-04

**Authors:** Ariel Rechtman, Tal Friedman-Korn, Omri Zveik, Lyne Shweiki, Garrick Hoichman, Adi Vaknin-Dembinsky

**Affiliations:** 1 Department of Neurology and Laboratory of Neuroimmunology and the Agnes-Ginges Center for Neurogenetics, Hadassah-Hebrew University Medical Center, Ein-Kerem, Germany; 2 Faculty of Medicine, Hebrew University of Jerusalem, Jerusalem, Israel; 3 Department of Military Medicine, Faculty of Medicine, Hebrew University of Jerusalem, Jerusalem, Israel; 4 Neurology Department, Multiple Sclerosis and Immunobiology Research, Hadassah Medical Center, Ein-Kerem, POB 12000, 91120 Jerusalem, Israel

**Correction to: Journal of Neurology** 10.1007/s00415-024-12438-6

In the original version of this article, author’s name Tal Friedman-Korn was incorrectly written as Tal Freidman‑Korn.

